# Illness Representation and Associated Factors among Hypertensive Patients in Central Ethiopia

**DOI:** 10.4314/ejhs.v32i2.10

**Published:** 2022-03

**Authors:** Daniel G/Tsadik, Yemane Berhane, Alemayehu Worku

**Affiliations:** 1 Department of Nursing, School of Health Sciences, Arsi University, Asella, Ethiopia; 2 Department of Epidemiology and Biostatistics, Addis Continental Institute of Public Health, Addis Ababa, Ethiopia; 3 School of Public Health, Addis Ababa University, Addis Ababa, Ethiopia

**Keywords:** Illness representation, illness perception, hypertension, Ethiopia

## Abstract

**Background:**

Illness representation is an implicit belief system about an illness constructed by an individual to give meaning to their illness. An individual's belief about his/her illness, treatment, and own control are known to influence an individual's ability to cope with the illness and sustain the health-related quality of life. However, how Ethiopians perceive hypertension has not been studied well. This study aimed to assess illness representation and associated factors among hypertensive patients in Central Ethiopia.

**Method:**

A facility-based cross-sectional study was conducted in four public hospitals in Central Ethiopia. A total of 989 patients participated in the study. The revised Illness Representation Questionnaire was used to collect relevant data. Data were analyzed using the Generalized Estimating Equation with an ordinal logistic regression model and exchangeable working correlation matrix. A P-Value of less than 0.05 was indicated statistical significance.

**Results:**

Overall, 64.3% (95% CI: 61.3, 67.4) of the respondents reported low to moderate Illness representation about their hypertension. Respondents who were housewife [AOR: 1.48, 95% CI= 1.05, 2.08], in older age category 50–64 years [AOR: 1.92, 95% CI= 1.19, 3.09] and ≥ 65 years [AOR: 2.38, 95% CI= 1.43, 3.96], and had no family support [AOR: 1.98, 95% CI= 1.44, 2.73] showed a significant association with Illness Representation.

**Conclusion:**

This study revealed that about two-thirds of hypertensive patients in Central Ethiopia perceived hypertension as a low to moderately threatening illness. Such low illness representation undermines initiation of treatment and effective control of blood pressure. Health care providers need to strengthen strategies that increase their patient's illness representation.

## Introduction

Illness Representation (IR) of hypertensive patients influences their coping behavior and the success of their blood pressure management (1). IR is theoretically defined as the person's efforts to organize, analyze, and interpret information about an illness and its symptoms including its identity, timeline, cause, controllability, and consequences (2). IR is the implicit belief system about an illness constructed by an individual to give meaning to their illness based on the information obtained from different sources (3). Patients assimilate lay information, personal experience, and information from the social environment to form an interpretation of the impact of the disease on their life (4). The acquired health information influences the patient's cognitive representations and emotional responses to the disease and illness experience (5).

The cognitive IRs have five constructs. These are identity (the label or symptoms an individual associates with the disease), cause (the individual's belief about the etiology of disease), timeline (the individual's belief about the duration of disease), consequences (belief about the impact of the disease on the individual's life) and cure/control (a belief about whether something can be done to recover from the illness) (6). On the other hand, Emotional representations are a psychological and subjective response to health threats and reflect their affective associations and responses to the cognitive-based IRs and lead to emotion-focused coping. There are two dimensions of emotional representations: Coherence (refers to the individual's degree of understanding or comprehension of the illness) and emotion (refers to the individual's affective feelings or response about the illness) (3,7).

IR is associated with treatment, adherence, and functional recovery and they form the basis for deciding which strategies, and behaviors patients will use to manage their illness. Highlevel IR can improve healthcare-seeking behavior and adherence to proper treatment. By understanding the IR of patients with hypertension; health care professionals can help patients adhere to the recommended lifestyle behaviors required for controlling high blood pressure (8). Previous studies identified factors such as gender (9), family history of hypertension (10), level of education (11), and income (12) to have a significant influence on illness representation.

IR is an important consideration for improving care for hypertensive patients. However, to our knowledge, IR among Ethiopian hypertensive patients has not been studied previously. Therefore, this research aimed to assess IR and associated factors among hypertensive patients in Central Ethiopia.

## Material and Methods

**Setting and participants**: The current study is part of a large study (Illness Representation, treatment adherence, and associated factors with it) conducted in Oromia regional state, central Ethiopia. A hospital-based cross-sectional study was conducted in four public hospitals; namely, Bishoftu, Adama, Asella, and Shashemene. These hospitals are located in Oromia regional state, central Ethiopia. These hospitals provide a comprehensive health care service for both outpatient and inpatient patients. The hospitals have specialty clinics where patients with specific chronic illnesses are referred for treatment and follow-up. The hypertension clinic is one of the specialty referral clinics in all study hospitals.

**Study population**: All adults receiving their antihypertensive treatment in the four study hospitals and who fulfilled the inclusion criteria participated in the study. The inclusion criteria include age 18 years or above, a diagnosis of hypertension confirmed by a treating physician, patients who have been on antihypertensive medication for at least 3 months, patients who can give consent to participate in the study, and patients who were not under any acute distress. Pregnant women were excluded from the study.

**Sample size and sampling procedures**: The sample size for determining the level of IR was calculated assuming a conservative illness representation prevalence of 50%, a 4% precision, and a 95% level of confidence. Thus, 600 patients were required for the study. For assessing factors associated with IR, we calculated the sample size based on the study conducted by Abere D. A. et.al. (13) using a stat-calc function of Epi Info statistical software considering the proportion of urban dwellers to be 28.7% among high IR group, OR= 1.55, 80% power and a ratio of 1:1. The calculated sample size was 1030, since this sample size was bigger than the former calculation, it is considered as a final sample size. Hypertensive patients who consented to participate in the study were consecutively enrolled until the required sample was fulfilled.

**Data collection tool and procedure**: Data were collected using a uniform and pre-tested questionnaire developed by reviewing different literature. The data collection tool contained questions on socio-demographic information, lifestyle, health-related matters, hypertension knowledge question (14), health beliefs, and illness perception (IPQ-R) (15). The hypertension evaluation of lifestyle and management (HELM) scale which has 11 items were used as a tool to assess respondents' knowledge (14). We used the validated IPQ-R (15) with seven subscales and 34 questions including the sub-dimensions (4 items of the timeline, 4 items of timeline cyclical, 6 items of consequences, 4 items of personal control, 5 items of treatment control, 5 items of illness coherence, and 6 items of emotional perceptions). In all dimension's subjects were given 5 options which were converted to a 5-point Likert type scale for analysis: Strongly Disagree (1), Disagree (2), Neither Agree nor Disagree (3), Agree (4), and Strongly Agree (5). The Hypertension belief and behavior questionnaire was developed based on the constructs of the Health Belief Model (HBM) (16). The HBM constructs include a 5-item Likert scale on perceived susceptibility (4 items), perceived severity (5 items), perceived benefits (5 items), perceived barriers (5 items), perceived self-efficacy (4 items), and cues to action (4 items).

Data were collected through a face-to-face interview by trained nurses, who were not routinely working at the hypertension clinics. The data collectors were competent to do interviews both in Amharic (the national language) and Afan Oromo (the state language). Additionally, one nurse was assigned to supervise the data collection process at each hospital.

Patients were interviewed after they got their routine services at the clinic exist in a private space. Before collecting data, interviewers explained the objective of the study and obtained informed verbal consent from each study participant.

**Statistical analysis**: The data were analyzed with SPSS (version 21.0). The mean (±SD) was reported for continuous variables, and the number (percentage) was presented for categorical variables. We also examined sociodemographic, health-related variables, and health belief items considering IR as an outcome variable. The correlation of the hospital's was taken into account using the Generalized Estimating Equation (GEE) with ordinal logistic regression model and exchangeable working correlation matrix. Initially, we select two subject variables or the combination of Hospital ID and Patient ID since patient identification numbers were not unique across hospitals. We select an ordinal logistic model to specify a distribution and link function as the dependent variable has three ordered levels IR (low, moderate, and high). The Exchangeable working correlation matrix (structure has homogenous correlations between elements) was applied to represent the within subject-subject dependencies during analysis. The variables which have a P-value of 0.25 or lower in the bivariate analysis were entered into multivariable ordinal logistic regression analysis. The GEE parameter estimates were expressed with their Odds ratio and the 95% confidence intervals (95% CI). A p-value< 0.05 was considered to indicate statistical s ignificance.

**The following operational definitions are used. Illness perception** is defined as patients' own implicit and common-sense beliefs about their illness. As the IPQ-R scores do not have a clinical cut-off (17), the total IR was grouped into three categories (low, moderate, and high) by tercile scores. Of the total scores, the score of 77 to 94 “low IR”, the score of 95 to 111 indicated “moderate IR” and 112 to 128 indicated “higher IR”

**Knowledge of hypertension** a total of eleven knowledge questions were used after reviewing from hypertension evaluation of lifestyle and management questionnaire. Each correct answer concerning the knowledge of hypertension was given one point and zero for incorrect answers. The total knowledge score of the respondents was varied between 0 (with no correct answer) and 11 (for all correct answers), and a cut-off level of <8 was considered as poor knowledge, and ≥8 indicated good knowledge.

**Ethical considerations**: Ethical approval was obtained from the Institutional Review Board of Arsi University. Support was sought from the Oromia Regional Health Bureau (ORHB) and Asella referral hospital. The ORHB provided support letters to Adama, Bishoftu, and Shashemene hospitals. Permission to conduct the study was subsequently obtained from the medical director's office of each study hospital. Each study participant was adequately informed about the purpose, method, anticipated benefit, and risk of the study by the data collectors. All interviews were conducted after obtaining consent from each participant. Interviews were conducted in a private space and confidentiality of the information was assured by removing identifiable information.

## Results

A total of 989 study participants were interviewed from four public hospitals in central Ethiopia, with a response rate of 96%. The mean (±SD) age of the participants was 57.6 ± 11.7 years while the majority (46.9%) belonged to 50–64 age groups and were mostly female (52.7%). The majority, 76.8%, of the study participants were urban residents, and married, 63.4%. About half (49.5%) of the participants had no formal education and the majority (64.9%) of the participants live within half an hour walking distance from the hospitals. (Table 1).

**Table 1:** Socio-demographic characteristics of hypertensive patients in Central Ethiopia 2017 (n=989)

Variable	Frequency	%
Sex		
Male	468	47.32
Female	521	52.68
Name of the hospital		
Asella	322	32.56
Adama	268	27.10
Bishoftu	246	24.87
Shashemen	153	15.47
Age		
20–34	29	2.9
35–49	198	20.0
50–64	464	46.9
65+	298	30.1
Mean and Sd (57.65		
+ 11.76)		
Residence		
Urban	760	76.85
Rural	229	23.15
Level of education		
No formal education	490	49.54
Primary (Grade 1–4)	177	17.90
Secondary (Grade 5–8)	172	17.39
Diploma & above	150	15.17
Marital status		
Never married	154	15.57
Married	627	63.40
Divorced	55	5.56
Widowed	153	15.47
Occupation		
GOV employed	154	15.57
Self-employed	151	15.27
Farmer	129	13.04
Housewife	325	32.86
Retired	230	23.3
Walking Distance of residence to hospital		
< an hour	642	64.9
≥ an hour	347	35.1

About half (54.4%) of the participants had been living with hypertension for one to five years. More than half of the patients (55.7%) visit the hospital once every month and 72.7% of the respondents had no family history of hypertension while more than three-quarters (79.1%) of them reported receiving support from their families (Table 2).

**Table 2:** Illness-related factors of hypertensive patients in Central Ethiopia 2017 (n=989)

Variable	Frequency	%
Duration of antihypertensive treatment		
< 5 years	538	54.4
≥ 5 years	451	45.6
Frequency of visit		
Once or less per month	633	64.0
Once in 2 months or more	356	36.0
Family history of hypertension		
Yes	234	23.7
No	755	76.3
Presence of family support		
Yes	782	79.1
No	207	20.9

More than half (56.6%) and 51.7% of the respondents took two or more antihypertensive drugs more than once per day, respectively. About three-fourths (75.1%) of them reported havinghad no side effects, however, 70.7% of them reported never being told about the side effects of the drugs by health care providers. Approximately half of the respondents (52.9%) had a comorbid illness and mostly (80.11%) reported illness was diabetes mellitus. Participants had a mean and standard deviation of systolic BP pressure of 139.46 mmHg (± 15.91) and diastolic BP of 86.34 mmHg (± 8.32). Fifty-eight percent of the respondents (n = 574) had recorded BP in the uncontrolled range at or above 140/90 mmHg (Table 3).

**Table 3:** Health-related conditions of hypertensive patients in Central Ethiopia, 2017 (n=989)

Variable	Frequency	%
Duration of antihypertensive treatment		
< 5 years	538	54.4
≥ 5 years	451	45.6
Number of antihypertensive drugs taken		
One drug	429	43.4
Two or more drugs	560	56.6
Antihypertensive drug dosage/frequency		
Once per day	477	48.23
More than once	512	51.77
Any history of side effects of antihypertensive drugs		
Yes	246	24.87
No	734	75.13
Ever told about the side effect of antihypertensive drugs		
Yes	290	29.32
No	699	70.68
Reported co-morbidity		
None	443	44.8
DM	419	42.4
Chronic kidney disease	33	6.3
Heart disease	45	8.6
Others	26	5.0
Anything that makes you stressed		
Yes	458	46.31
No	531	53.69

We have assessed hypertensive patients' beliefs using the health belief model constructs. The findings indicated that 59.5% of the respondents had lower beliefs on the risk of contracting an illness or its complications (perceived susceptibility), 52.4% of the respondents showed lower beliefs that potential factors might make it difficult to take the behavior (perceived barriers), and 56.9% of the respondents reported lower internal or external cues that prompt the action. Whereas 65.7% of them reported higher beliefs that the disease is severe and has serious consequences (perceived severity), 77.1% reported higher beliefs that taking treatment is beneficial to reduce or prevent disease threat (perceived benefit), and 51.7% of them had relatively good confidence to tackle their illness (self-efficacy) (Table 4).

**Table 4:** Hypertension belief and behavior of patients in Central Ethiopia, 2017 (n=989)

Variable	Frequency	%
Perceived susceptibility		
Low	588	59.5
High	401	40.5
Perceived severity		
Low	339	34.3
High	650	65.7
Perceived benefits		
Low	226	22.9
High	763	77.1
Perceived barriers		
Low	518	52.4
High	471	47.6
Cues to action		
Low	563	56.9
High	426	43.1
Self-efficacy		
Low	478	48.3
High	511	51.7

Concerning the patients IR, 35.7% (95%CI: 32.5, 38.5) of the respondents showed lower IR, 33.7% (95% CI: 30.7, 36.7%) moderate IR and 30.6% (95% CI: 27.9, 33.8) higher IR.

Overall, the minimum and maximum scores of the respondents for IR, items were between 77 and 128 with a Mean and standard deviation of 104.70 (± 8.57). The seven dimensions of IR were also assessed. The mean and SD for timeline whether an illness is acute or chronic 13.9 (±1.59), for the consequences 20.4(±2.89), treatment control 15.7(±1.94), personal control (±1.87), illness coherence 13.2(±2.18), cyclical 11.1(±3.05) and emotional representation 17.9(±4.34%) (Table 5).

**Table 5:** IR of hypertensive patients in Central Ethiopia, 2017 (n=989)

Illness representation	Score Mean
Items	(SD)*
Timeline acute/chronic	13.19 ± 1.59
Consequence	20.44 ± 2.84
Treatment control	15.74 ± 1.94
Personal control	13.14 ± 1.87
Illness coherence	13.21 ± 2.18
Cyclical coherence	11.05 ± 3.05
Emotional representation	17.91 ± 4.34
Overall IR	104.70+ 8.57

* SD= Standard Deviation

**Factors associated with IR: multivariable GEE analysis**: After controlling possible confounding effects of other covariates four factors remained as significant independent predictors of IR in the GEE ordinal logistic regression model. These are Age of the patients, Marital status, occupation and family support. Respondents who were never married [AOR: 0.12, 95% CI= 0.65, 0.23], married respondents [AOR: 0.20, 95% CI= 0.11, 0.38], being a housewife [AOR: 1.48, 95% CI= 1.05, 2.08], age of the respondents 50–64 years [AOR: 1.92, 95% CI= 1.19, 3.09], ≥ 65 years [AOR: 2.38, 95% CI= 1.43, 3.96] and respondents who had family support [AOR: 1.98, 95% CI= 1.44, 2.73] showed a significant association with IR (Table 6).

**Table 6:** Multivariable analysis for associations between IR and covariates, performed by GEE with Ordinal logistic regression among hypertensive patients in Central Ethiopia, 2017. (n =989)

	Illness representations			AOR (95%CI)
		
Variables	High	Moderate	Low	
Sex of the patients				
Male	150	162	156	Ref
Female	153	171	153	0.91 (0.71, 1.17)
Patients' residence				
Urban	213	253	294	Ref
Rural	90	80	59	1.22 (0.89, 1.66)
Educational status of patients				
No formal education	161	172	157	Ref
Primary	57	55	65	1.15 (0.72, 1.43)
Secondary	52	65	55	1.17 (0.83, 1.64)
Diploma & above	33	41	76	0.78 (0.54, 1.12)
Marital status of the patients				
Never married	41	56	57	0.12 (0.65, 0.23)**
Married	202	215	210	0.20 (0.11, 0.38)**
Divorced	19	16	20	1.45 (0.53, 3.83)
Widowed	41	46	66	Ref
Occupation of patient				
Government employed	40	63	51	1.14 (0.75, 1.73)
Self-employed	48	48	55	1.03 (0.70, 1.52)
Farmer	33	44	52	0.98 (0.66, 1.45)
Housewife	122	101	102	1.48 (1.05, 2.08)*
Retired	60	77	93	Ref
Age of patient				
20–34	6	13	10	Ref
35–49	56	65	77	1.05 (0.86, 1.29)
50–64	143	150	171	1.92 (1.19, 3.09)**
≥ 65	98	105	95	2.38 (1.43, 3.96)**
Presence of family support				
Yes	231	265	286	1.98 (1.44, 2.73)**
No	72	68	67	Ref

* Statistically Significant (**P<0.05**)

** high statistically significant (**P < 0.001**)

## Discussion

The finding of this research showed nearly two-thirds of the respondents had low to moderate IR. A lower Illness Representation was more likely among older patients, housewives, and those who lack family support.

Understanding individuals' perceptions about their illnesses provide essential for developing effective strategies to manage chronic illnesses such as hypertension. The findings of this study indicated about two-thirds of the respondents reported low to moderate IR. This finding is in agreement with previous studies on illness perception among patients with hypertension (18, 19); the majority of patients in low-income settings perceive hypertension as a benign illness that had minimal effect on their lives.

The low to moderate IR we observed in this study might be due to a low level of knowledge on hypertension among our study population; about two-thirds of our study participants having poor knowledge. Also, as hypertension is largely asymptomatic (20), patients might not feel any ill health effects, which leads to low perceived coherence (21, 22). The low IR can negatively influence their illness-related behaviors, patients may have poor adherence to treatments and lose control of their blood pressure. Poor adherence and control of hypertension might increase the risk of developing serious complications like stroke and heart attack leading to serious disability and even death (23).

Our result indicates age is significantly associated with illness representation, as age increases the respondent's IR decreases. This finding concurred with a few other studies that show an association between age and IR (24,25) in which younger patients have more threatening illness perception (24). Another possible explanation could be as age increases comorbid illnesses become more common (26). This could make it difficult for individuals to ascribe specific symptoms to specific diseases (27) and therefore could lead to overlapping representations of multiple illnesses (28). On the contrary, a study was done by Sawicki GS. et al, 2011 found that younger patients with hypertension have a more benign illness perception (25). We also noted that some studies did not find demographic factors, especially age, to be associated with a person's illness perception (9, 29).

The finding of this research revealed respondents having strong perceived family support showed a significant association with IR towards hypertension. This finding corroborates the fact that Africans have a naturally rich social support network, family-centered societies, and are likely to get meaningful support from family members (30,31). Similarly, married patients are likely to get better family support than their unmarried counterparts (32).

In conclusion, about two-thirds of the hypertensive patients showed a low to moderate IR; thus perceived hypertension as a low to moderate threatening illness. As this is a major hurdle to initiating treatment and achieving reasonable control. Therefore, health care providers need to design appropriate interventions to improve illness representation for improving the management of hypertension and thereby prevent the burden of complications caused by uncontrolled hypertension.
